# The emerging role of ubiquitin-specific protease 20 in tumorigenesis and cancer therapeutics

**DOI:** 10.1038/s41419-022-04853-2

**Published:** 2022-05-04

**Authors:** Qiong Li, Chanqi Ye, Tian Tian, Qi Jiang, Peng Zhao, Xiaoting Wang, Feiye Liu, Jianzhen Shan, Jian Ruan

**Affiliations:** 1 Department of Medical Oncology, The First Affiliated Hospital, Zhejiang University School of Medicine, Hangzhou, 310003 Zhejiang Province People’s Republic of China; 2 Department of Medical Oncology, The First Affiliated Hospital, Zhejiang University School of Medicine, Key Laboratory of Cancer Prevention and Intervention, Ministry of Education, Hangzhou, Zhejiang Province People’s Republic of China; 3 Department of Oncology, Integrated hospital of traditional Chinese Medicine, Southern Medical University, Guangzhou, 510315 Guangdong People’s Republic of China

**Keywords:** Targeted therapies, Ubiquitylation

## Abstract

As a critical member of the ubiquitin-specific proteolytic enzyme family, ubiquitin-specific peptidase 20 (USP20) regulates the stability of proteins via multiple signaling pathways. In addition, USP20 upregulation is associated with various cellular biological processes, such as cell cycle progression, proliferation, migration, and invasion. Emerging studies have revealed the pivotal role of USP20 in the tumorigenesis of various cancer types, such as breast cancer, colon cancer, lung cancer, gastric cancer and adult T cell leukemia. In our review, we highlight the different mechanisms of USP20 in various tumor types and demonstrate that USP20 regulates the stability of multiple proteins. Therefore, regulating the activity of USP20 is a novel tumor treatment. However, the clinical significance of USP20 in cancer treatment merits more evidence. Finally, different prospects exist for the continued research focus of USP20.

## Facts

- USP20 is upregulated in multiple cancer types.
- USP20 mainly exhibits a promotive effect in breast cancer, lung cancer, and colon cancer; however, it shows inhibitory effects in gastric cancer.
- Regulating the expression of USP20 is a novel treatment for tumors.
- Deletions of USP20 exhibit an inhibitory effect on the tumorigenesis of some cancer types.

## Open questions

- Does USP20 have a common mechanism in tumors?
- Can these emerging inhibitors of USP20 be successfully applied soon in clinical practice?

## Introduction

**Fig. 1:**
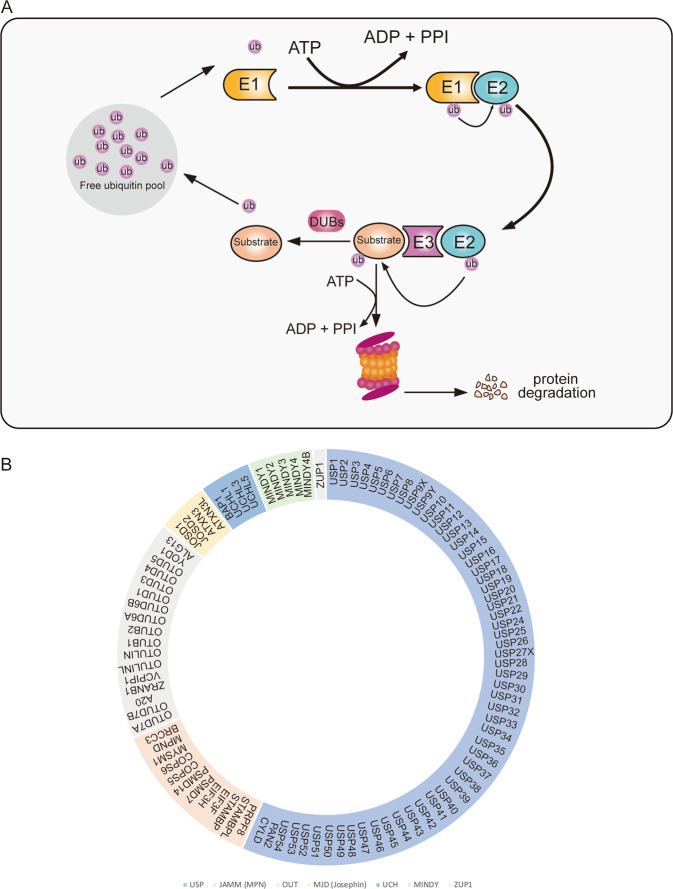
The ubiquitin–proteasome system cascade and the classification of deubiquitylase family. **A** Diagrammatic of key events in ubiquitylation and deubiquitylation. The E1 enzyme activates and combines with ubiquitin moiety in an ATP-dependent manner. Then the ubiquitin moiety is transferred to an E2 conjugating enzyme, Finally, ubiquitin is transferred directly from E2 enzyme to substrate protein by E3 ligase, on the one hand, the labeled protein is degraded by the 26s proteasome. Or the DUBs stabilize the targeted protein by removing the ubiquitin moiety, the ubiquitin becomes free ubiquitin to reuse. **B** The reported subclass of DUBs including ubiquitin-specific proteases (USPs), ubiquitin C-terminal hydrolases (UCHs), ovarian tumor proteases (OTUs), JAMMs (also known as MPN+), MJDs (also known as Josephins), and the two new families: MINDY family and the ZUP1 family.

The ubiquitin–proteasome system (UPS) is the most important post-transcriptional modification in eukaryotic cells [1, 2]. The components of this system include ubiquitin (ub), E1 ubiquitin-activating enzyme, E2 ubiquitin-conjugating enzyme, E3 ubiquitin ligase and 26S proteasome [3]. Ubiquitylation is a reversible posttranslational modification that plays a role in various biological processes, including protein degradation [4], DNA damage and repair [5], cell cycle progression [6], and immune response [7]. Ubiquitin, comprising 76 amino acids, is highly conserved among eukaryotes and contains seven lysine residues, K6, K11, K27, K29, K33, K48, and K63 [8]. Of these residues, K48 regulates the degradation of target proteins by linking polyubiquitin, K63 increases cell signal transduction and protein kinase activation by linking linear polyubiquitin chains, and the others are linked by mono- or polyubiquitin chains [9]. The UPS includes two steps. First, in ubiquitylation, one or more ubiquitin proteins are added to tag the substrate proteins. Second, the marked proteins will be identified by the 26S proteasome for cleavage, degradation, and recycling [10]. Deubiquitylation is the opposite process of ubiquitylation. Ubiquitination and deubiquitylation are always in a state of dynamic equilibrium [10]. Deubiquitinating enzymes (DUBs) are involved in deubiquitylation, which can rescue the marked substrate proteins by remodeling and removing conjugated ubiquitin chains [11] (Fig. 1A). The balance between ubiquitin enzymes and DUBs ultimately determines the ubiquitination status of a given target protein, making protein ubiquitination a multifunctional and dynamic posttranslational modification. Ubiquitination plays a key role in multiple cellular processes, including gene expression [12], cell cycle progression [13], DNA damage and repair [14], cell growth [15], and apoptosis [16]. These ubiquitination-regulated processes are critical to maintain cellular homeostasis, and abnormal regulation of these processes contributes to tumor development [17, 18]. The importance of ubiquitination in cancer-related cell function and successful use of the proteasome inhibitor bortezomib in multiple myeloma have attracted increased attention concerning the potential of ubiquitination/deubiquitination proteins in tumor therapy [19, 20].

Recently, an increasing number of studies have demonstrated that DUBs regulate various biological or pathological processes by stabilizing tumor or antitumor proteins [21–24]. As recently described, human DUBs are subdivided into seven families, ubiquitin-specific proteases (USPs), ubiquitin C-terminal hydrolases (UCHs), ovarian tumor proteases (OTUs), JAMMs (also known as MPN+ and hereafter referred to as JAMM/MPN+), MJDs (also known as Josephins), the MINDY family and the ZUP1 family (these two new families of DUBs were discovered recently) [25] (Fig. 1B). Among them, USPs are the largest subfamily of DUBs that participate in the progression of multiple tumors [26–29]. Therefore, studies on DUBs as a therapeutic target warrant further exploration. Table 1 shows detailed information about different types of DUBs and their roles in different cancers. Presently, many related inhibitors of DUBs have been used in tumor therapeutic models, further illustrating the potential of DUBs as therapeutic targets. Previous studies have described that ubiquitin-specific peptidase 20 (USP20) plays a critical role in tumorigenesis [30]. In this article, we first briefly discuss the function of USP20, focusing on its increasingly recognized potential as a target in cancer treatment. Finally, we summarize the alterations of USP20 in multiple human cancers and discuss novel findings regarding the potential of this enzyme as a tumor therapeutic.

**Table 1:** Other DUBs and cancer.

DUB	Cancer type	Tumorigenesis	Reference
USP1	Osteosarcoma	Promotes the invasion of osteosarcoma cells	[84]
	Glioma	USP1 stabilizes EZH2 to activate β-catenin to drive glioma tumorigenesis	[85]
	Breast cancer	Regulates metastasis	[86]
USP2	Breast cancer	Promotes cell migration and invasion	[87]
USP4	Breast cancer	Promotes invasion	[88]
	Melanoma	May be an oncogene	[89]
	Glioblastoma	Promotes glioblastoma multiforme	[90]
		Facilitates chemoresistance	[91]
USP5	Pancreatic cancer	Promotes tumorigenesis and progression	[92]
	Non-small cell lung cancer	Upregulation of USP5 contributes to tumorigenesis	[93]
	Colorectal cancer	Promotes cell growth and resistance to chemotherapeutics	[94]
USP7	Medulloblastoma	Promotes medulloblastoma cell survival and metastasis	[95]
	Breast cancer	Promotes breast carcinogenesis via stabilizing PHF8	[96]
	Osteosarcoma	Promotes metastasis by inducing EMT	[97]
	Lung cancer	Modulates the antitumor immune response	[98]
USP8	Cervical cancer	Associated with a poor prognosis in cervical squamous cell carcinoma patients	[99]
		Suppresses apoptosis by stabilizing FLIP_L_	[100]
USP10	Colon cancer	Promotes tumor proliferation	[101]
	Lung cancer	Inhibits cell proliferation, invasion and cell growth	[102, 103]
		The USP10-HDAC6 confers cisplatin resistance	[104]
	Liver cancer	Promotes cell proliferation by stabilizing YAP/TAZ	[105]
		Promotes metastasis by stabilizing Smad4	[106]
		Inhibits p53 Signaling and constricts poor outcome	[107]
	Acute myeloid leukemia	Stabilizes oncogenic FLT3	[108]
USP11	Colorectal cancer	Promotes growth and metastasis	[109]
	Ovarian cancer	Promotes EMT by stabilizing Snail	[110]
USP13	Non-small cell lung cancer	Promotes tumor progression	[111]
USP14	Breast cancer	Regulates cell cycle progression and cell proliferation and metastasis	[112–114]
		Enhances sensitivity to enzalutamide	[115]
USP30	Liver cancer	Stabilizes DRP1 to promote hepatocarcinogenesis	[116]
UCHL1	Gastric cancer	Promotes metastasis	[117]
OTUB2	Non-small cell lung cancer	Promotes tumorigenesis	[118]

## Structure and function of USP20

**Fig. 2:**
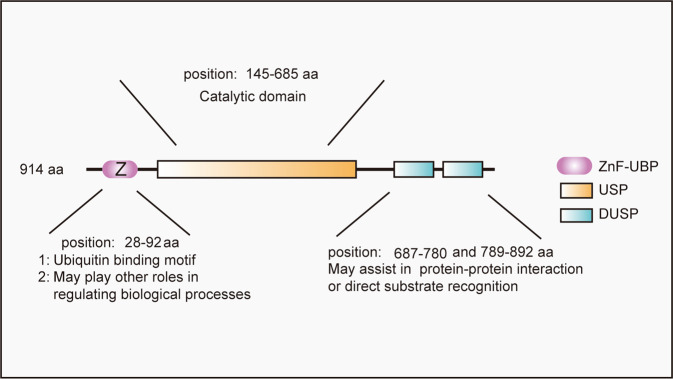
The structure of USP20 and the function of each domain of USP20. USP20 contains three different domains, including ZNF-UBP, USP, and DUSP domain. Those different domains of USP20 play crucial roles in different biological processes.

The ubiquitin-specific proteolytic enzyme family is the largest subtype of DUBs identified thus far. USP20, a specific member of this family, is also called pVHL-interacting deubiquitinating enzyme 2 (VDU2) [31, 32], and was first identified as a von Hippel–Lindau (VHL) syndrome-related deubiquitinase [33, 34]. Human USP20 comprises 914 amino acids with a predicted molecular weight of 102 kDa [34]. The primary structure of human USP20 includes an N-terminal zinc finger ubiquitin-specific protease (ZnF-UBP) domain, a USP catalytic domain and two tandem domain present in ubiquitin-specific proteases (DUSP) domains [35] (Fig. 2). The ZnF-UBP domain is also found in some other USPs and is the ubiquitin-binding motif [36]. However, the definite function of this domain of USP20 has not been demonstrated very clearly until now. Yang et al. demonstrated that the ZnF-UBP domain of USP20 presented weak binding capacity to monoubiquitin [34], however, this domain characteristically binds with K48-linked di-ubiquitin [37]. The USP catalytic domain, the structure of which is similar to other members of the USP family, is the most important functional area of USPs and exhibits strong homology in two regions that surround the catalytic Cys box and His box [38]. USP20 comprises three domains that form a shape that looks like the right hand extended. These three domains are similar to the “palm”, “finger” and “thumb” domains. This right-hand-like structure can form a ubiquitin-binding surface that is convenient for ubiquitin binding. The catalytic center is located between the “palm” and “thumb”, and the “hand” holds the ubiquitin molecule of the target proteins. Next, the ubiquitin molecules are removed from the labeled proteins so that the ubiquitin molecules and proteins are recycled and reused [39, 40]. The DUSP domain is a tripod structure similar to AB3 formed by three alpha-helices forming a bundle structure to support three strands of antiparallel beta folds [35]. Studies have reported that the DUSP domain in DUBs may play a crucial role in protein–protein interactions or direct substrate recognition [35]. Kommaddi et al. demonstrated that the phosphorylation status at serine 333 of USP20 is critical for deubiquitinase activity. Subsequently, they found that protein kinase A phosphorylates USP20 on serine 333, inhibits the trafficking of the substrate β2AR (β2 adrenergic receptors) and decreases degradation via autophagosomes [41]. Berthouze et al. also demonstrated that USP20 served as a novel regulator for β2AR recycling and resensitization [42]. Lu et al. demonstrated that mechanistic target of rapamycin complex 1 phosphorylated USP20 at serine 132 and serine 134 and then increased the stability of HMG-CoA reductase (HMGCR), the rate-limiting enzyme in the cholesterol biosynthetic pathway [43].

## USP20 as a potential cancer target

**Fig. 3:**
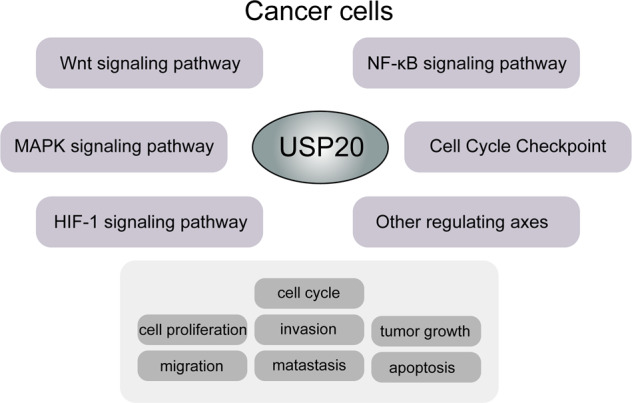
The mechanism of deubiquitinating enzyme USP20 in cancer. USP20 regulates cell proliferation, migration, invasion, metastasis, and tumor growth by participating in multiple signaling pathways in various cancer types.

As a member of the largest subfamily of DUBs, USP20 plays key roles in various tumors by stabilizing tumorigenic or antitumor proteins. USP20 was first identified as a substrate of the VHL tumor suppressor protein [32]. The pVHL protein is encoded by the VHL gene, which plays a key role in cellular oxygen sensing by ubiquitinating hypoxia-inducing factors and is then degraded by the proteasome [44]. The dysregulation of the pVHL protein is related to VHL (Hippel–Lindau syndrome) disease. This disease is a rare autosomal dominant hereditary tumor syndrome involving multiple systems, manifested as multiple familial benign and malignant tumors and cysts of the central nervous system and internal organs [45–47]. Considering that USP20 is a substrate of the pVHL protein, we speculate that pVHL protein inactivation induces decreased degradation of USP20, promoting the progression of VHL disease. An additional characterized substrate of the pVHL E3 ligase complex is the α-subunit of hypoxia-inducible factor 1 (HIF1α) [48]. Early studies have reported that HIF1α is overexpressed in various cancer types and regulates the expression of most genes involved in many essential biological and pathological processes [49–52]. Li et al. found that USP20 recognizes and binds to HIF1α, removes its ubiquitin chain, and maintains high expression levels of HIF1α, increasing the transcription of hypoxia-inducible element genes [48]. Considering that HIF1α activates the transcription of genes that are involved in crucial aspects of cancer biology, including angiogenesis [53], cell survival [54], glucose metabolism [55], and invasion [56], by triggering multiple signaling pathways, USP20 can regulate multiple biological processes by stabilizing tumorigenic or antitumor proteins. In addition, studies have demonstrated that USP20 triggers the activation of multiple pathways, including Wnt, MAPK, HIF1, NF-κB, cell cycle checkpoint and many other signaling pathways [33, 57–60], promoting the processes of multiple cancer types (Fig. 3). As a results, these findings prompt USP20 may be a potential cancer target.

## Role of USP20 in several types of cancer

### Colon cancer

Colon cancer is a leading cause of cancer-related death worldwide [61]. Wu et al. first identified β-catenin as a substrate of USP20, and their study also suggested that USP20 stabilizes β-catenin by decreasing the ubiquitination of β-catenin in vivo and in vitro [57]. To further investigate the role of the USP20-β-catenin axis in cancers, they detected the protein and mRNA levels of USP20 and β-catenin in colon cancer and other cancer cell lines, including osteosarcoma, cervical, breast, ovarian, and colorectal cancer cell lines [57]. They demonstrated that USP20 and β-catenin were upregulated and correlated in most of these cancer cell lines. And they also observed that USP20 overexpression markedly increased cell proliferation, migration and invasion in these cancer cell lines. They demonstrated that USP20 expression was positively correlated with the β-catenin levels in clinical colon cancer samples [57]. Previous publications have identified β-catenin as an oncogene [62], prompting us to speculate that USP20 promotes tumorigenesis by regulating β-catenin stability. Thus, the USP20-β-catenin axis may be an emerging therapeutic target in colon cancer.

### Breast cancer

Breast cancer is the leading cause of cancer-related death among women [61]. Sowa et al. first identified USP20 as a candidate interacting with the extracellular signal-regulated kinase 3 (ERK3) protein via global proteomics analysis [63]. Mathien et al. found that USP20 was regulated in breast cancer and that the discovered USP20 played a crucial role in the migration of breast cancer lines [58]. Among the mechanisms by which USP20 affects the migration of breast cancer cell lines, they demonstrated that USP20 was correlated with ERK3, and the stability of ERK3 protein was increased by USP20 [58]. And they investigated the possible effect of USP20 on the contribution of ERK3. They demonstrated that USP20 overexpression could enhance HeLa cell migration, and they further examined the impact of the USP20-ERK3 axis in regulating the migration of breast cancer cell lines [58]. First, their studies showed that both USP20 and ERK3 are overexpressed in multiple breast cancer cell lines, including MCF7, T47D, and SKBR3. They observed a strong significant correlation between the USP20 and ERK3 protein levels. Second, they depleted USP20 in MCF7 cell lines, resulting in markedly decreased ERK3 protein levels and reduced migration of MCF7 cells [58]. These results indicate that the USP20-ERK3 axis can promote the migration of breast cancer cell lines, suggesting that USP20 may play an important role in the tumorigenesis of breast cancer.

Another important regulating mechanism in breast cancer is the USP20-SNAI2 axis. SNAI2 (also known as SLUG) is reported as a metastasis-related transcription factor [64]. Previous studies have shown that estrogen receptor α (ERα) repressed the expression of SNAI2 [65], and the protein level of ERα correlated inversely with SNAI2 in breast cancer cell lines and tissues [66]. Li et al. first identified SNAI2 as one substrate of USP20, they found that USP20 can increase the stability of SNAI2, and subsequently increase the migration and invasion of ER^–^ breast cancer cell lines [67]. In an in vitro study, they found that knockdown of USP20 can suppress the lung colonization of breast cancer cell lines. Meanwhile, they detected USP20 and SNAI2 in ER^–^ clinical breast cancer samples, and demonstrated that USP20 positively correlated with SNAI2. Higher protein level of USP20 and SNAI2 was also demonstrated to predict worse prognosis in ER^–^ breast cancer patients [67]. Thus, their study suggested that USP20-SNAI2 axis may serve as a novel therapeutic target axis in breast cancer.

### Cervical cancer

Cervical cancer has been a cause of cancer-related death in recent years among women [61]. Ha et al. first demonstrated that USP20 plays a critical role in regulating the stability of p62 in tumor necrosis factor (TNFα)-mediated nuclear factor kappa light chain enhancer of activated B cells (NF-κB) activation [59]. In their study, they found that USP20 increased the stability of p62 by deubiquitinating lysine 48 (K48)-linked polyubiquitination, promoting cell survival. They further demonstrated the depletion of UPS20 in HeLa cervical cancer cell lines, resulting in a reduced NF-κB-mediated pro-survival signal and increased receptor-interacting serine/threonine protein kinase 1 (RIPK1)-independent apoptosis [59]. Their data defined a novel mechanism by which USP20 regulates the protein level of p62. They found that a high level of p62 protein promoted cell survival and decreased the cell death of HeLa cell lines [59]. This report is the first to reveal the role of the USP20-p62 axis in NF-κB-mediated cell survival. This finding suggests that USP20 acts as an essential regulator in cancer progression and will be a novel therapeutic target for cancer.

Autophagy is critical in tumorigenesis in multiple cancer types [68–70]. Autophagy begins with the formation of autophagosomes, a process that depends on the activity of the serine/threonine kinase ULK1 (hATG1) [71]. A previous study reported that USP20 is localized to the endoplasmic reticulum (ER) [72, 73], and USP20 may play an important role in autophagy. Kim et al. demonstrated that USP20 acts as a positive regulator of the autophagy process by increasing the stability of unc51-like kinase 1 (ULK1) in the HeLa cell line [74]. They found that this regulation also existed in the colon cancer cell lines HCT116 and HT29, and the basal level of ULK1 was the critical factor in inducing autophagy initiation [74]. In their study, they also reported USP20 dissociated from ULK1 at a later time after autophagy induction and promoted the next step in autophagy. The dissociated ULK1 transited into lysosomes to degrade and maintain the basal level. They also found that USP20 is critical for cell survival under starvation [74]. Dynamic regulation of the USP20-ULK1 axis may act as a promising target to inhibit autophagy in certain human diseases.

### Gastric cancer

Wang et al. verified that both USP20 and Claspin proteins are expressed at low levels in human gastric cancer tissues, and this phenomenon was also observed in gastric cancer cell lines (MGC-803, NCI-N87, MKN45, BGC-823, KATO III, SGC-7901, AGS, SNU-1, SNU-16 and MKN74) [60]. They also found that low USP20 expression levels correlated with a poor prognosis in patients [60]. In their study, they demonstrated USP20 regulated the stability of Claspin and thus modulating the activation of the cell cycle checkpoint. Low expression of USP20 significantly promoted cell proliferation and accelerated the transition from G1 to S phase of the cell cycle [60]. This finding suggested that USP20 could inhibit cell proliferation of gastric cancer cells by regulating Claspin. It prompted us to speculate that USP20 is a promising new molecular target to design new therapeutic modalities to control the development and progression of gastric cancer.

### Adult T cell leukemia

Adult T cell leukemia (ATL) is a fatal hematopoietic malignant tumor caused by type 1 human T cell leukemia virus (HTLV-1) infection [75–77]. Yasunaga et al. first identified USP20 as the first DUB shown to deubiquitinate Tax, and their findings also suggested that ubiquitinated Tax was necessary for activation of the NF-κB signaling pathway [78]. Tax is expressed in many ATL cell lines [77]. Interestingly, Yasunaga et al. found that USP20 expression was significantly low in these cell lines [78]. Because NF-κB is a pro-survival and pro-proliferative factor in HTLV-1 cells, they discovered that the upregulation of USP20 protein negatively regulated NF-κB signal transduction and inhibited cell proliferation of the ATL2 cell line [78]. A previous study showed that ubiquitinated TNF receptor associated factor 6 (TRAF6) is a key regulator of the activation of the NF-κB signaling pathway [79]. Yasunaga et al. also found that Tax overexpression increases TRAF6 ubiquitination, activating NF-κB. TRAF6 ubiquitination induced by Tax was also sensitive to USP20 deubiquitination [78]. In summary, their findings demonstrated that USP20 acted as a DUB for Tax and TRAF6 and as a negative regulator of NF-κB signal transduction. These results prompted us to speculate that USP20 may be a potential new therapeutic target for ATL.

## Challenges and prospects

**Fig. 4:**
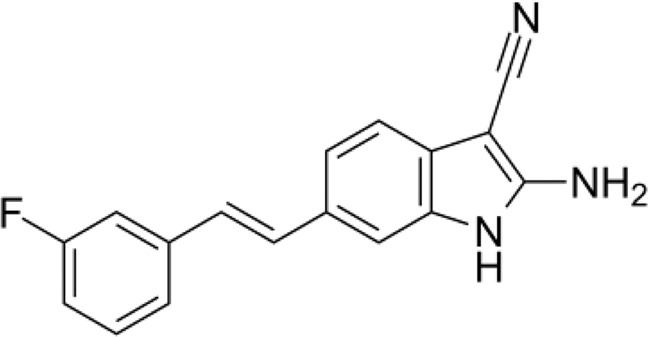
The structure of GSK2643943A. The structure of GSK2643943A, one small molecule inhibitor for USP20/Ub-Rho.

In the past few years, researchers have overcome many difficulties and have screened many small-molecule compounds, including inhibitors and activators. Table 2 presents many preclinical inhibitors of DUBs. However, only one small-molecule compound exists for USP20. GSK2643943A, a small-molecule inhibitor targeting USP20/Ub-Rho, was first identified by GlaxoSmithKline (GSK) from a screen involving compounds. The structure of this USP20 inhibitor is shown in Fig. 4, but this inhibitor is still in the preclinical stage [80, 81]. Lu et al. demonstrated that mammalian target of rapamycin complex 1 phosphorylates USP20 at S132 and S134 and then stabilizes HMGCR, increasing cholesterol biosynthesis in the liver [43]. And their study also showed genetic deletion or pharmacological inhibition of USP20 markedly decreased diet-induced body weight gain and reduced lipid levels in the serum and liver [43]. Although no DUB inhibitors are in ongoing clinical trials, with the rapid development of inhibitors, DUBs are likely increasingly attractive as drug targets. USP20, as a member of the largest deubiquitinase family, was first identified as an oncogene [30]. Although USP20 attracts our attention as a potential therapeutic target, it is associated with challenges. The first challenge is to develop DUB inhibitors or activators, including the development of USP20 inhibitors or activators. On the one hand, the mechanisms of function of DUB enzymes are usually complicated and involve regulating enzyme activity through allosteric and/or substrate-mediated catalysis. Many DUBs dynamically change between active and inactive conformations [81, 82]. On the other hand, because most DUBs perform ubiquitin transfer via reactive thiol groups, most standard assays used to identify inhibitors are nonselective redox or alkylation false-positives [83]. Second, to date, only one USP20 inhibitor has been identified. Although Lu et al. found that this USP20 inhibitor, GSK2643943A, inhibits the function of USP20 to improve metabolic-related diseases [43], no data are available in tumor-related studies. The lack of preclinical research data will be one of the biggest challenges for the transfer of USP20 inhibitors to clinical applications. Third, the mechanism by which USP20 promotes tumor progression in this review has been elucidated in a few types of cancer cells, but the mechanism is unclear in most cancer cell lines that highly express USP20. However, a common mechanism in different types of tumors should also be a challenge for transferring the inhibitor to clinical applications. We require more evidence to support USP20 as a potential tumor therapeutic target or combine USP20 inhibitors with other drugs in cancer treatment. Fourth, presently, the impact of USP20 on tumor development occurs only in cells or animal models, and clinical samples must be further verified. Fifth, through searching currently available literature, we have summarized the role of USP20 in the regulation of cellular proliferation, migration, tumor growth, and metastasis. Although USP20 serves as an oncogene in varied cancer types, it also acts as a tumor suppressor in a few cancer types, including ATL and gastric cancer. The different roles of USP20 may attribute to the heterogeneity of different tumor types. Specifically, different from the observation in cervical cancer that USP20 regulates the stability of p62 in TNFα mediated NF-κB activation, USP20 act as a DUB that negatively regulates NF-κB signal transduction in ATL. Moreover, it regulates the cell cycle checkpoint activation in gastric cancer. In this respect, more studies are needed to further elucidate the function of USP20 and the underlying mechanism in diverse malignancies. Screening activators for USP20 as a therapeutic alternative is also promising for malignancies with low USP20 levels. However, USP20 as a tumor therapeutic target has its limitations. We believe that USP20 as a tumor therapy target is expected to become a reality in the clinic with the in-depth exploration of the role of USP20 in tumor progression.

**Table 2:** Other representative inhibitors of DUBs.

DUB inhibitor	Target	Structure	Stage of development	IC50	Reference
SJB3-019A	USP1		Preclinical	78.1 nM	[119]
ML323	USP1		Preclinical	76 nM	[120]
ML364	USP2		Preclinical	1.1 μM	[121]
P5091	USP7		Preclinical	4.2 μM	[122, 123]
FT671	USP7		Preclinical	52 nM	[124]
GNE-6776	USP7		Preclinical	N.A.	[125]
DUBs-IN-1	USP8		Preclinical	0.24 μM	[126]
Spautin1	USP13 and USP10		Preclinical	0.6–0.7 μM	[127, 128]
Mitoxantrone	USP11		Preclinical	8.5 μM	[129]
IU1	USP14		Preclinical	4–5 μM	[130]
IU1-47	USP14		Preclinical	0.6 μM	[131]
IU1-248	USP14		Preclinical	0.83 μM	[132]
PR619	Broad range DUB inhibitor		Preclinical		[133, 134]
1,10-Phenanthroline	JAMM type Isopeptidase		Preclinical		[135]
VLX1570	USP14 and UCHL5		Clinical trial phase (now suspended)	10 μM	[136]
GSK2643943A	USP20/Ub-Rho		Preclinical	160 nM	[43]

## Conclusion

In this summary, USP20, a DUB belonging to USPs, is responsible for removing ubiquitin moieties from ubiquitin-labeled proteins. An increasing number of researchers have focused on exploring the function of USP20 in regulating tumorigenesis. Recently, many breakthroughs have been made in clarifying the role of USP20 in regulating cell proliferation, migration, tumor growth, and glucose metabolism by regulating different signaling pathways. These results also provide evidence for our speculation that USP20 is a target for tumor therapy. More importantly, GSK screened one inhibitor of USP20 and showed that this inhibitor affects the expression of USP33, which shares approximately 59% identity with USP20 and has strong homology at the amino and carboxy termini. Therefore, novel USP20 inhibitors may provide potential treatment options for USP20-overexpressing cancer types. Further analysis of the molecular signaling pathway of USP20 can offer new insights into its tumorigenesis or antitumor mechanisms. The specific regulation of tumorigenesis by USP20 may be a hot research topic in the future. The development of clinical drugs for USP20 will also provide a new opportunity for tumor treatment.
